# Blood Pressure and Systemic Risk Factors in Patients Presenting With Post‐Extraction and Periodontal Bleeding at a Dental Out‐of‐Hours Clinic

**DOI:** 10.1002/cre2.70446

**Published:** 2026-08-26

**Authors:** Yosuke Iijima, Miki Yamada, Shunsuke Hino, Motohiko Sano, Norio Horie, Takahiro Kaneko

**Affiliations:** ^1^ Department of Oral and Maxillofacial Surgery, Saitama Medical Center Saitama Medical University Kawagoe Saitama Japan; ^2^ Division of Applied Pharmaceutical Education and Research Hoshi University Tokyo Japan

**Keywords:** antithrombotic, blood pressure, diabetes mellitus, hypertension, periodontal disease‐related gingival bleeding, post‐extraction bleeding

## Abstract

**Objective:**

This study aimed to verify whether blood pressure was higher in patients presenting to the dental out‐of‐hours clinic with post‐extraction bleeding (PEB) and periodontal disease‐related gingival bleeding (PDB) compared to those presenting for other conditions (Control (C) group). Additionally, we examined differences in the prevalence of hypertension, antithrombotic use, and diabetes mellitus among the PEB, PDB, and C groups.

**Materials and Methods:**

Patients visiting the out‐of‐hours clinic were retrospectively categorized by sex and age (young (Y)‐group: 20–39, middle (M)‐group: 40–59, older (O)‐group: ≥ 60). Data collected included systolic (SBP) and diastolic (DBP) blood pressure, and the prevalence of treated hypertension, antithrombotic use, and diabetes mellitus. Mean BP values were compared using Welch's *t*‐test. The prevalence of systemic factors was compared using Fisher's exact test. Multivariable logistic regression analyses were performed in the O‐group.

**Results:**

A total of 416 bleeding patients were included, comprising 216 patients with PEB, 119 with PDB, and 81 with other bleeding. Because of the limited number of cases in the Y and M‐groups, multivariable logistic regression analyses were performed only in the O‐group (≥ 60 years). According to that, antithrombotic use was independently associated with both PDB and PEB in males and females. Increased SBP was independently associated with PDB in both sexes and with PEB in males. Hypertension remained independently associated with PDB only in females, whereas diabetes mellitus was not independently associated with either bleeding condition.

**Conclusion:**

Particularly among the O‐group (≥ 60 years), antithrombotic use was independently associated with both PDB and PEB, whereas increased systolic blood pressure was independently associated with PDB and, to a lesser extent, PEB. These findings highlight the importance of systemic assessment, particularly regarding blood pressure and antithrombotic therapy, in patients presenting with acute oral bleeding.

## 1 Introduction

The dental and oral surgery out‐of‐hours clinic treats patients presenting with various dental and oral surgical conditions requiring urgent attention. In our previous report, we noted that blood pressure readings for patients attending the dental and oral surgery out‐of‐hours clinic were higher than those observed in the national survey (Iijima et al. 2025a; National Health and Nutrition Survey 2019). This suggests that patients attending these out‐of‐hours clinics are experiencing significant stress due to the underlying condition presenting as their chief complaint or systemic factors. Patients presenting with intraoral bleeding frequently attend out‐of‐hours clinics, and we have often observed empirically that among those attending dental and oral surgery out‐of‐hours clinics, patients presenting with bleeding as their chief complaint tend to have elevated blood pressure.

This suggests that even among patients attending the out‐of‐hours clinic, blood pressure readings may vary depending on the disease category, particularly among those presenting with bleeding as their chief complaint. It is thought that the proportion of hypertension, which is the elevation of blood pressure itself, may also differ.

The most common types of bleeding encountered in the out‐of‐hours clinic are post‐extraction bleeding (PEB) and periodontal disease‐related gingival bleeding (PDB) (Iijima et al. 2025b). Numerous reports indicate that PEB is strongly associated with the use of antithrombotic agents. The incidence of PEB was reported as 0.3% in the non‐antithrombotic therapy group, 0.6% in the single antiplatelet therapy group, 1.3% in the dual antiplatelet therapy group, 1.7% in the Direct Oral Anticoagulant (DOAC) group, 1.9% in the warfarin group, and 2.8% in the combined antiplatelet and anticoagulant therapy group (Nakamura et al. 2024). Compared with those who experienced no bleeding after tooth extraction, patients with PEB were significantly more likely to have hypertension, alongside those with kidney disease, liver disease, and blood coagulation disorders (Nakamura et al. 2024). Regarding PDB, Iijima et al. report that it is prevalent among out‐of‐hours emergency attendees, particularly those aged 60 and over, with a significantly higher incidence among hypertensive patients. Locally, it is considered to be strongly associated with periodontal disease (Iijima et al. 2025b).

It has been reported that patients with hypertension have a higher incidence of hemorrhagic disorders such as cerebral hemorrhage (Li et al. 2017). These findings suggest that patients presenting to the out‐of‐hours clinic with bleeding as their chief complaint may have higher blood pressure than those attending for other conditions, and may have a higher proportion of hypertension. If elevated blood pressure is indicated, the patient must be instructed to maintain appropriate blood pressure levels.

This study aimed to verify whether blood pressure was higher in patients presenting to the dental out‐of‐hours clinic with bleeding (PEB and PDB) as their primary complaint compared to those presenting for other conditions (Control group). Additionally, we examined the differences in the prevalence of hypertension, antithrombotic use, and diabetes mellitus among the PEB, PDB, and Control groups.

## 2 Materials and Methods

This retrospective study was approved by the Research Ethics Committee of Saitama Medical Center, Saitama Medical University (reference number: 2025‐033).

### 2.1 Study Population

Patients aged 20 years or older who visited the out‐of‐hours oral surgery clinic at Saitama Medical Center, Saitama Medical University between February 19, 2013 and January 31, 2025 with bleeding as the chief complaint, specifically those presenting for PEB or PDB. The out‐of‐hours outpatient clinic operates from 17:30 to 8:30 the following day. PEB was defined as bleeding occurring solely after uncomplicated tooth extraction, excluding bleeding following other dental surgical procedures. PDB was defined as sudden, spontaneous bleeding confined to the gingival sulcus, excluding bleeding immediately following periodontal treatment such as scaling (Iijima et al. 2025b). Exclusion criteria: Patients whose bleeding was primarily caused by severe systemic factors such as overt coagulation factor abnormalities (e.g., hemophilia) or hematologic malignancies (e.g., leukemia) were excluded. Cases where the electronic medical records lacked sufficient documentation for research purposes were also excluded. The control group mainly consisted of patients presenting with non‐bleeding dental emergencies, including temporomandibular joint dislocation and other non‐hemorrhagic oral conditions.

### 2.2 Data Collection

The following data were collected: systolic blood pressure (SBP) and diastolic blood pressure (DBP) at presentation, along with the presence of hypertension and diabetes currently under treatment, and antithrombotic use. Blood pressure measurements were obtained in the out‐of‐hours examination room prior to the doctor's visit, using either an electronic sphygmomanometer or manual sphygmomanometer by a nurse.

### 2.3 Data Analysis

The collected data was categorized by sex and age group: 20–39 years old (young group, Y‐group), 40–59 years old (middle group, M‐group), 60 years old and above (older group, O‐group), and compared with data from the control group (C group).

### 2.4 Statistical Analysis

Continuous variables (SBP and DBP) are presented as the mean ± standard deviation. Categorical variables (hypertension, antithrombotic use, and diabetes mellitus) are presented as counts and percentages.

#### 2.4.1 Blood Pressure Comparison

Mean SBP and DBP values among the PEB, PDB, and Control (C) groups within each age and sex category were compared using Welch's *t*‐test.

#### 2.4.2 Systemic Factor Comparison

The prevalence of hypertension, antithrombotic use, and diabetes mellitus between groups was compared using Fisher's exact test.

#### 2.4.3 Multivariable Analysis

Multivariable logistic regression analyses were performed. Separate models were constructed for males and females. The dependent variable was the presence of PDB or PEB, and the independent variables included SBP, hypertension, antithrombotic use, and diabetes mellitus. Although SBP and hypertension are clinically related, both variables were included in the multivariable models because they represent different clinical concepts: SBP reflects the actual blood pressure measured at presentation, whereas hypertension represents a medical history of hypertension currently under treatment. Odds ratios (ORs) and 95% confidence intervals (CIs) were calculated.

All statistical tests were two‐sided, and a *p*‐value < 0.05 was considered statistically significant. All statistical tests were two‐sided, and a *p*‐value < 0.05 was considered statistically significant. Statistical analyses were performed using SPSS 25.0 for Windows (IBM Corp., Armonk, NY).

## 3 Results

The study population comprised a total of 416 bleeding patients: 216 with PEB (110 males, 106 females), 119 with PDB (64 males, 55 females), and 81 with other bleeding (44 males, 37 females). Other bleeding included such as bleeding following dental surgical procedures (20 males, 14 females) and bleeding from tumors (6 males, 7 females). The age distribution of participants is detailed in Table 1.

**Table 1:** Demographic and age distribution of study participants.

	Male				Female			
Age group (Year)	Post‐extraction bleeding (PEB)	Periodontal disease‐related gingival bleeding (PDB)	Other bleeding	Control (C)	Post‐extraction bleeding (PEB)	Periodontal disease‐related gingival bleeding (PDB)	Other bleeding	Control (C)
Young (Y) (20 ~ 39)	*n* = 39	*n* = 3	*n* = 10	*n* = 440	*n* = 32	*n* = 1	*n* = 5	*n* = 283
Middle (M) (40 ~ 59)	*n* = 22	*n* = 7	*n* = 10	*n* = 362	*n* = 30	*n* = 8	*n* = 8	*n* = 290
Older (O) (≥ 60)	*n* = 49	*n* = 54	*n* = 24	*n* = 286	*n* = 44	*n* = 46	*n* = 24	*n* = 364

*Note:* Other bleeding includes bleeding following dental surgical procedures, bleeding following scaling, and bleeding from tumors.

Among the Y and M‐groups, the number of cases in the bleeding groups was limited, particularly in the Y‐group with PDB. Therefore, multiple comparisons and multivariable analyses were not performed in these age groups. Nevertheless, except for the Y‐group with PDB, both PEB and PDB in male and female groups generally tended to have higher SBP values than the control group. In the M and O‐groups, the bleeding groups also tended to show a higher prevalence of hypertension and a higher frequency of antithrombotic use than controls (Tables 2A, 2B and Table 3A, 3B, 3C).

**Table 2A:** Comparison of systolic and diastolic blood pressure in male patients.

	Systolic blood pressure (mean ± standard deviation)			Diastolic blood pressure (mean ± standard deviation)		
Age group (Year)	Post‐extraction bleeding (PEB)	Periodontal disease‐related gingival bleeding (PDB)	Control (C)	Post‐extraction bleeding (PEB)	Periodontal disease‐related gingival bleeding (PDB)	Control (C)
Young (Y) (20 ~ 39)	128.3 ± 20.3 (*n* = 39)	112.7 ± 10.3 (*n* = 3)	125.5 ± 15.5 (*n* = 440)	80.1 ± 16.5 (*n* = 39)	82.3 ± 14.6 (*n* = 3)	74.5 ± 13.2 (*n* = 440)
Middle (M) (40 ~ 59)	142.8 ± 23.7 (*n* = 22)	150 ± 16.6 (*n* = 7)	138.4 ± 19.6 (*n* = 362)	86.2 ± 24.5 (*n* = 22)	99.3 ± 16.0 (*n* = 7)	86.6 ± 14.2 (*n* = 362)
Older (O) (≥ 60)	150.8 ± 23.8 (*n* = 49)	152.6 ± 26.4 (*n* = 54)	145.3 ± 25.3 (*n* = 286)	85.9 ± 14.5 (*n* = 49)	87.0 ± 17.3 (*n* = 54)	85.6 ± 17.1 (*n* = 286)

**Table 2B:** Comparison of systolic and diastolic blood pressure in female patients.

	Systolic blood pressure (mean ± standard deviation)			Diastolic blood pressure (mean ± standard deviation)		
Age group (Year)	Post‐extraction bleeding (PEB)	Periodontal disease‐related gingival bleeding (PDB)	Control (C)	Post‐extraction bleeding (PEB)	Periodontal disease‐related gingival bleeding (PDB)	Control (C)
Young (Y) (20 ~ 39)	121.4 ± 13.9 (*n* = 32)	99 (*n* = 1)	119.4 ± 14.8 (*n* = 283)	74.8 ± 10.7 (*n* = 32)	57 (*n* = 1)	73.7 ± 12.6 (*n* = 283)
Middle (M) (40 ~ 59)	139.4 ± 25.3 (*n* = 30)	163 ± 40.4 (*n* = 8)	133.8 ± 21.7 (*n* = 290)	81.4 ± 16.9 (*n* = 30)	92.3 ± 19.7 (*n* = 8)	80.1 ± 15.1 (*n* = 290)
Older (O) (≥ 60)	153.3 ± 26.6 (*n* = 44)	159.4 ± 21.4 (*n* = 46)	147.5 ± 25.3 (*n* = 364)	81.0 ± 15.8 (*n* = 44)	85.0 ± 16.1 (*n* = 46)	80.4 ± 15.3 (*n* = 364)

**Table 3A:** Association with hypertension prevalence.

	Male *n* (%)			Female *n* (%)		
Age group (Year)	Post‐extraction bleeding (PEB)	Periodontal disease‐related gingival bleeding (PDB)	Control (C)	Post‐extraction bleeding (PEB)	Periodontal disease‐related gingival bleeding (PDB)	Control (C)
Young (Y) (20 ~ 39)	0 (0.0%)	0 (0.0%)	5 (1.1%)	1 (3.1%)	0 (0.0%)	3 (1.1%)
Middle (M) (40 ~ 59)	4 (18.2%)	3 (42.9%)	61 (16.9%)	6 (20.0%)	4 (50.0%)	26 (9.0%)
Older (O) (≥ 60)	32 (65.3%)	39(75.0%)	134 (46.6%)	27 (64.7%)	35 (72.0%)	145 (41.4%)

**Table 3B:** Association with antithrombotic use prevalence.

	Male *n* (%)			Female *n* (%)		
Age group (Year)	Post‐extraction bleeding (PEB)	Periodontal disease‐related gingival bleeding (PDB)	Control (C)	Post‐extraction bleeding (PEB)	Periodontal disease‐related gingival bleeding (PDB)	Control (C)
Young (Y) (20 ~ 39)	0 (0.0%)	0 (0.0%)	1 (0.3%)	1 (2.3%)	0 (0.0%)	0 (0.0%)
Middle (M) (40 ~ 59)	2 (4.1%)	4 (7.4%)	16 (5.6%)	2 (4.5%)	2 (4.3%)	7 (1.9%)
Older (O) (≥ 60)	36 (73.5%)	38 (70.4%)	105 (36.7%)	26 (59.1%)	24 (52.2%)	60 (16.5%)

**Table 3C:** Association with diabetes mellitus prevalence.

	Male *n* (%)			Female *n* (%)		
Age group (Year)	Post‐extraction bleeding (PEB)	Periodontal disease‐related gingival bleeding (PDB)	Control (C)	Post‐extraction bleeding (PEB)	Periodontal disease‐related gingival bleeding (PDB)	Control (C)
Young (Y) (20 ~ 39)	0 (0.0%)	0 (0.0%)	2 (0.7%)	0 (0.0%)	0 (0.0%)	1 (0.3%)
Middle (M) (40 ~ 59)	2 (4.1%)	2 (3.7%)	15 (5.2%)	1 (2.3%)	2 (4.3%)	14 (3.8%)
Older (O) (≥ 60)	10 (20.4%)	13 (24.1%)	54 (18.9%)	10 (22.7%)	6 (13.0%)	53 (14.6%)

In the O‐group, multiple comparisons were performed. Among older male patients (Table 4A), SBP was significantly higher in the PDB group than in the control group. The prevalence of hypertension was significantly higher in the PDB group than in controls, whereas antithrombotic use was significantly more frequent in both the PEB and PDB groups than in controls. No significant differences were observed for diabetes mellitus. Among older female patients (Table 4B), SBP was significantly higher in the PDB group than in the control group. Hypertension was significantly more prevalent in both the PEB and PDB groups than in controls, and antithrombotic use was also significantly more frequent in both bleeding groups. No significant differences were observed for diabetes mellitus.

**Table 4A:** Comparison of systemic factors among older male patients.

Variables	PEB (*n* = 49)	PDB (*n* = 54)	Control (*n* = 286)	Significant pairwise comparison (Bonferroni‐adjusted *p* value)
SBP (mean ± SD mmHg)	150.8 ± 23.8	152.6 ± 26.4	143.3 ± 22.9	PDB vs. Control: ***p*** = **0.021**
DBP (mean ± SD mmHg)	85.8 ± 14.4	87.0 ± 17.3	81.1 ± 15.1	None
Hypertension, *n* (%)	32 (65.3)	39 (72.2)	134 (46.9)	PDB vs. Control: ***p*** = **0.0019**
Antithrombotic use, *n* (%)	36 (73.5)	38 (70.4)	105 (36.7)	PEB vs. Control: ***p*** < **0.0001**
				PDB vs. Control: ***p*** < **0.0001**
Diabetes mellitus, *n* (%)	10 (20.4)	13 (24.1)	54 (18.9)	None

*Note:* Bold values are statistically significant.

Abbreviations: DBP, diastolic blood pressure; PDB, periodontal disease‐related gingival bleeding; PEB, post‐extraction bleeding; SBP, systolic blood pressure; SD, standard deviation.

**Table 4B:** Comparison of systemic factors among older female patients.

Variables	PEB (*n* = 49)	PDB (*n* = 54)	Control (*n* = 286)	Significant pairwise comparison (Bonferroni‐adjusted *p* value)
SBP (mean ± SD mmHg)	153.3 ± 26.6	159.4 ± 21.3	147.5 ± 25.3	PDB vs Control: ***p*** = **0.0027**
DBP (mean ± SD mmHg)	81.0 ± 15.8	85.0 ± 16.1	80.4 ± 15.3	None
Hypertension, *n* (%)	27 (61.4)	35 (76.1)	145 (39.8)	PEB vs Control: ***p*** = **0.0273**
				PDB vs Control: ***p*** < **0.0001**
Antithrombotic use, *n* (%)	26 (59.1)	24 (52.2)	60 (16.5)	PEB vs. Control: ***p*** < **0.0001**
				PDB vs. Control: ***p*** < **0.0001**
Diabetes mellitus, *n* (%)	10 (22.7)	6 (13.0)	53 (14.6)	None

*Note:* Bold values are statistically significant.

Abbreviations: DBP, diastolic blood pressure; PDB, periodontal disease‐related gingival bleeding; PEB, post‐extraction bleeding; SBP, systolic blood pressure; SD, standard deviation.

Multivariable logistic regression analyses were performed in the O‐group to identify factors independently associated with PDB and PEB (Tables 5A and 5B).

**Table 5A:** Multivariable logistic regression analysis for factors associated with PDB in older patients.

Variable	Male OR (95% CI)	*p* value	Female OR (95% CI)	*p* value
SBP (per 1 mmHg increase)	1.015 (1.002–1.027)	**0.022**	1.017 (1.005–1.030)	**0.006**
Hypertension	1.91 (0.96–3.79)	0.065	3.38 (1.58–7.23)	**0.0017**
Antithrombotic use	3.32 (1.71–6.44)	**< 0.001**	4.42 (2.21–8.86)	**< 0.001**
Diabetes mellitus	1.15 (0.55–2.41)	0.701	0.54 (0.20–1.43)	0.217

*Note:* Bold values are statistically significant.

Abbreviations: CI, confidence interval; OR, odds ratio; PDB, periodontal disease‐related gingival bleeding; SBP, systolic blood pressure.

**Table 5B:** Multivariable logistic regression analysis for factors associated with PEB in older patients.

Variable	Male OR (95% CI)	*p* value	Female OR (95% CI)	*p* value
SBP (per 1 mmHg increase)	1.013 (1.000–1.027)	**0.048**	1.012 (0.999–1.024)	0.067
Hypertension	1.31 (066–2.61)	0.436	1.24 (0.60–2.59)	0.558
Antithrombotic use	4.47 (2.19–9.12)	**< 0.001**	7.19 (3.49–14.80)	**< 0.001**
Diabetes mellitus	0.94 (0.42–2.08)	0.873	1.06 (0.45–2.47)	0.896

*Note:* Bold values are statistically significant.

Abbreviations: CI, confidence interval; OR, odds ratio; PEB, post‐extraction bleeding; SBP, systolic blood pressure.

For PDB (Tables 5A), antithrombotic use was independently associated with PDB in both males (OR 3.32, 95% CI 1.71–6.44, *p* < 0.001) and females (OR 4.42, 95% CI 2.21–8.86, *p* < 0.001). Increased SBP was also independently associated with PDB in both males (OR 1.015, 95% CI 1.002–1.027, *p* = 0.022) and females (OR 1.017, 95% CI 1.005–1.030, *p* = 0.006). Hypertension remained independently associated with PDB only in females (OR 3.38, 95% CI 1.58–7.23, *p* = 0.0017). Diabetes mellitus was not significantly associated with PDB in either sex.

For PEB (Table 5B), antithrombotic use was independently associated with PEB in both males (OR 4.47, 95% CI 2.19–9.12, *p* < 0.001) and females (OR 7.19, 95% CI 3.49–14.80, *p* < 0.001). Increased SBP remained independently associated with PEB only in males (OR 1.013, 95% CI 1.000–1.027, *p* = 0.048). Neither hypertension nor diabetes mellitus showed significant independent associations with PEB in either sex.

## 4 Discussion

This study compared blood pressure and the prevalence of systemic risk factors in patients presenting to the dental out‐of‐hours clinic for bleeding conditions, specifically PEB and PDB, against a control group (C).

Because the number of cases in the Y and M‐groups was limited, statistical testing was restricted to the O‐group. Nevertheless, except for the Y‐group with PDB, both PEB and PDB groups generally tended to have higher SBP values than the control group. In the bleeding groups also tended to show a higher prevalence of hypertension and a higher frequency of antithrombotic use than controls.

Among older patients, antithrombotic use was independently associated with both PDB and PEB in males and females. Increased SBP was independently associated with PDB in both sexes and with PEB in males.

The higher prevalence of PDB in the O‐group is primarily attributed to the age‐related progression of periodontal disease, which serves as a local prerequisite for bleeding (Eke et al. 2016).

Blood pressure naturally rises with age, mainly due to aortic stiffening and plaque accumulation (Benetos 2002). High blood pressure is a potent and primary risk factor for major hemorrhagic disorders, such as intracerebral hemorrhage (Li et al. 2017), and contributes to retinal hemorrhage and epistaxis (Biecker 2013; Byun et al. 2020; Del Pinto et al. 2020).

While PDB is clearly a manifestation of chronic periodontal disease, PEB is also frequently linked to pre‐existing periodontitis, which is a leading indication for tooth extraction in older adults (Eke et al. 2016). When significant periodontal disease is present prior to extraction, the surrounding periodontal tissues and microvasculature are already compromised and fragile due to chronic inflammation. Therefore, PEB can also be considered a form of bleeding significantly influenced by the underlying periodontal status, merely exacerbated by the surgical trauma of the extraction procedure. Given that the surgical wound encompasses the entire socket circumference, the bleeding pattern of PEB differs inherently from the spontaneous bleeding of PDB (Iijima et al. 2025b).

The elevated blood pressure and significantly higher prevalence of hypertension observed in both the PEB and PDB groups indicate that these acute oral bleeding events share common vascular‐related systemic characteristics. Furthermore, multivariable analyses demonstrated that increased SBP remained independently associated with PDB in both males and females and with PEB in males. In contrast, hypertension itself remained independently associated only with PDB in females. These findings suggest that actual blood pressure at presentation may be a more important indicator of bleeding risk than a previous diagnosis of hypertension alone. The fact that PDB patients presented with the highest blood pressure suggests that PDB is a clinical manifestation highly correlated with poorly controlled systemic vascular health.

Although the specific causality between hypertension and periodontal disease is still debated (Del Pinto et al. 2020; Sanz et al. 2020), hypertension may exacerbate gingival bleeding by stressing the gingival microvasculature and increasing hemorrhagic tendency in inflamed tissues (Ozmeric et al. 2024). Conversely, chronic inflammation from periodontitis may contribute to systemic vascular dysfunction, thereby elevating blood pressure (Del Pinto et al. 2020; Sanz et al. 2020).

Consistent with previous reports (Bhandari et al. 2025), antithrombotic use increased with age and was more prevalent among patients presenting with oral bleeding. Importantly, multivariable logistic regression analysis demonstrated that antithrombotic use remained independently associated with both PDB and PEB in males and females after adjustment for blood pressure, hypertension, and diabetes mellitus. While antithrombotic use is a well‐established factor associated with post‐extraction bleeding, the present findings indicate that it is also independently associated with periodontal disease‐related gingival bleeding. These findings suggest that antithrombotic therapy contributes not only to postoperative bleeding but also to spontaneous periodontal bleeding requiring emergency dental care. Considering the dramatically increased risk of major bleeding for individuals combining hypertension with anticoagulants (Li et al. 2017), the frequent observation of PDB in patients with both high blood pressure and antithrombotic use highlights a critical population for emergent bleeding management. Future research should assess the synergistic effect between elevated blood pressure and antithrombotic use on oral bleeding.

Diabetes mellitus is a major systemic disease crucial for comprehensive dental management, given its association with microvascular complications and impaired wound healing. Diabetes mellitus is strongly linked to periodontitis via a bidirectional association (Stöhr et al. 2021). The mechanisms by which diabetes mellitus potentially exacerbates gingival bleeding include increased inflammatory cytokines, impaired white blood cell function, and vascular fragility caused by microangiopathy (Sanz et al. 2018).

Despite this recognized link, our study found no consistent trend in diabetes mellitus prevalence across the PEB, PDB, and C groups. Furthermore, diabetes mellitus was not independently associated with either PDB or PEB in the multivariable analyses. This outcome, consistent with previous local reports (Iijima et al. 2025b), suggests that diabetes mellitus may not be a dominant factor leading to the presentation of acute, chief‐complaint bleeding (PEB or PDB) at an emergency clinic. While diabetes mellitus is critical for long‐term periodontal health, its role in immediate, acute oral bleeding presentation appears less pronounced in this setting compared to hypertension or antithrombotic use.

The limitations of this single‐institution retrospective study, including the small sample size for certain subgroups and the inability to assess renal and hepatic diseases, necessitate future multi‐center studies with larger sample sizes. Although this study was limited to older adults, we were able to identify the characteristics of patients' background factors, as the dental out‐of‐hours clinic with acute oral bleeding is more common among this population.

In conclusion, among older patients, antithrombotic use was independently associated with both PDB and PEB in males and females, indicating that it represents a common systemic factor associated with emergency oral bleeding presentations. Elevated SBP was also independently associated with PDB in both sexes and with PEB in males. In contrast, diabetes mellitus was not independently associated with either condition. These findings highlight the importance of comprehensive systemic assessment, particularly regarding blood pressure control and antithrombotic therapy, in patients presenting with acute oral bleeding.

## Author Contributions

**Yosuke Iijima:** conceptualization, writing – original draft. **Shunsuke Hino:** data curation, writing – original draft. **Motohiko Sano** and **Miki Yamada:** data curation supervision, writing – review and editing. **Norio Horie:** data curation, supervision, writing – review and editing. **Takahiro Kaneko:** conceptualization, project administration, supervision, writing – review and editing.

## Funding

The authors have no nothing to report.

## Consent

Written informed consent was obtained from the patient to publish this report in accordance with the journal's patient consent policy.

## Conflicts of Interest

The authors declare no conflicts of interest.

## Data Availability

The data that support the findings of this study are available from the corresponding author upon reasonable request.
